# Efficacy analysis of non-invasive correction for congenital cryptotia with auricular cartilage deformities

**DOI:** 10.1016/j.bjorl.2025.101630

**Published:** 2025-05-05

**Authors:** Zhiying Zhou, Jiahua Shen, Lexi Lin, Wenxin Chen, Yong Fu

**Affiliations:** Zhejiang University School of Medicine, Children's Hospital, Department of Otolaryngology, Head and Neck Surgery, Hangzhou, China

**Keywords:** Cryptotia, Cartilage deformity, Non-invasive correction, 3-months-old

## Abstract

**Objectives:**

To investigate the efficacy of non-invasive correction for congenital cryptotia with auricular cartilage deformities in infants.

**Methods:**

A total of 42 cases of congenital cryptotia with auricular cartilage deformities treated with non-invasive auricular correctors in our hospital from January 2019 to December 2023 were collected. The enrolled children were divided into four groups (A: less than 28-days, B: 28–90 days, C: 90–180 days, D: more than 180-days) based on age. The effective rate, treatment time, complications, and recurrence rate of non-invasive auricular correction among the four groups were compared.

**Results:**

After treatment with non-invasive auricular correctors, all four groups of children showed 100% effectiveness. The upper part of the children's auricles that were embedded in the temporal subcutaneous tissue improved compared to before correction, with high improvement rates in groups A and B for the correction of cartilage deformities, and the auricular shape basically returned to normal appearance after cartilage correction. In group D, the embedded part improved compared to before correction, but there was no significant change in cartilage deformities. Regarding treatment time, there was no statistical difference between groups B and C (*p* > 0.05), but there were statistical differences among the other groups. In terms of complications, groups A, B, C, and D showed statistical differences (*p* < 0.05). In terms of recurrence rate, after six months of observation, no cases of the auricular cartilage being re-embedded under the skin or cartilage deformities recurring after correction were observed in any of the four groups.

**Conclusion:**

The effect of non-invasive correction for congenital cryptotia with auricular cartilage deformities in infants is significant, with shorter correction times, higher correction rates for cartilage deformities, and fewer complications for those under three months old; older children can still consider non-invasive correction, but the correction time is relatively longer, and for those over six months old, the correction is still effective, but the rate of correction for cartilage deformities is lower, and there are more complications.

## Highlights

• Non-invasive correction effective for congenital cryptotia with cartilage deformities.
• Shorter correction time for infants under 3-months with high cartilage deformity rate.
• Non-invasive correction considered for infants over 3-months, longer duration needed.
• Correction still effective for infants over 6-months, lower cartilage deformity rate.

## Introduction

**Fig. 1:**
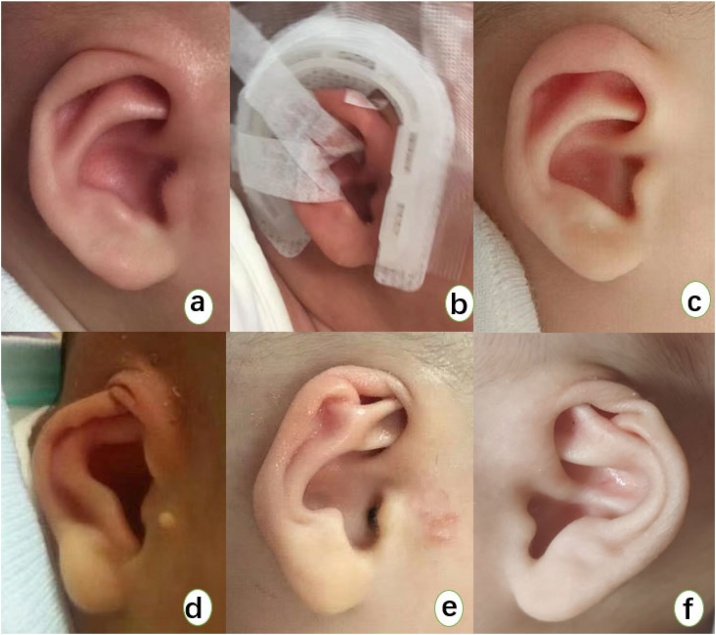
(Figures a‒c) Show the correction process of a 3-month-old infant with mild cryptotia without auricular cartilage deformity. Figure a shows the pre-correction appearance, with the upper part of the auricular cartilage buried under the temporal skin. (Figure b) Shows the correction process of cryptotia without cartilage deformity. (Figure c) Shows the appearance after correction with an auricular molding device for 3-weeks. (Figures d‒f) Show three cases of cryptotia with cartilage deformity. In addition to the upper part of the auricular cartilage being buried under the temporal skin and insufficient skin at the upper pole of the auricle, these cases also present with cartilage adhesion and/or cartilage hypoplasia.

Cryptotia is a relatively common congenital auricular deformity, mainly characterized by the upper part of the auricle being buried under the temporal skin with the absence of the superior auricular sulcus.^1^, ^2^, ^3^ From the perspective of auricular cartilage deformity, the main issue with congenital cryptotia is the folding and adhesion of the auricular cartilage; when the upper part of the auricle is manually lifted upwards, the entire auricle can be revealed.^4^ In addition to affecting appearance, cryptotia also causes inconvenience in daily life for children, as they cannot wear glasses and masks due to the upper part of the auricle being buried under the skin without an auricular sulcus. Furthermore, during bathing or getting wet in the rain, the lack of auricular protection allows water to directly enter the ear canal, potentially causing otological diseases such as otitis externa.^5^ The early classification of cryptotia was mainly based on the morphology of the antihelix and the state of the auricular muscles during surgery, dividing cryptotia into the helical muscle type (antihelix upper foot type) and the oblique muscle type (antihelix lower foot type).^6^ Some scholars also categorize cryptotia into types I and II based on whether the upper pole of the auricle is buried, and further divide them into subtypes O, A, B, and C according to the characteristics of auricular cartilage adhesion, which is relatively complex for clinicians.^7^ Professor Zhang Tianyu and his team in China have categorized cryptotia deformities into three degrees based on whether they are combined with cartilage adhesion deformity and cartilage dysplasia, according to previous classification standards: Degree I cryptotia is characterized by skin deficiency at the upper pole of the auricle; Degree II cryptotia features insufficient skin at the upper pole of the auricle with cartilage adhesion; Degree III cryptotia is characterized by insufficient skin at the upper pole of the auricle with cartilage dysplasia.^3^ In recent years, with the use of non-invasive auricular correctors, many studies have reported that mild cryptotia, which does not involve auricular cartilage deformity, has a significant effect with non-invasive correction (Fig. 1). Moreover, treatment within 6-months of age still yields noticeable results.^8^, ^9^, ^10^ However, there is currently a lack of research on whether non-invasive correction is feasible for cryptotia with cartilage deformity (Fig. 1), and most clinicians still believe that it is not suitable for children with congenital cryptotia and cartilage deformity to undergo non-invasive correction, and they should wait for later plastic surgery. This article, based on previous research on non-invasive treatment of cryptotia, collected clinical data of cases treated with non-invasive auricular correctors for congenital cryptotia with cartilage deformity in our hospital over the past 5-years, and studied the efficacy of non-invasive correction for infants and young children with congenital cryptotia with cartilage deformity.

## Methods

### Inclusion criteria

Cryptotia with cartilage deformity: The main characteristics include the upper half of the auricle being buried under the temporal scalp, absence of the superior auricular sulcus, deficiency or insufficiency of skin at the upper pole of the auricle, with or without cartilage adhesion, and accompanied by underdeveloped auricular cartilage.

### Clinical data

Forty-eight cases of congenital cryptotia with cartilage deformity treated with non-invasive auricular correctors in our hospital from January 2019 to December 2023 were selected, involving 42 children, with 29 ears on the right side and 19 ears on the left side. Among them, there were 27 boys and 15 girls, with an age range of 18–330 days at birth, and an average of 85.94 days. All children in the study were full-term births and passed the neonatal hearing screening. None of the children had purulent discharge from the ear canal or severe eczema, and they were in good general health.

### Experimental groups

The included children were divided into four groups based on their age: A (<28 days), B (28–90 days), C (90–180 days), and D (>180 days). The efficacy, treatment time, complications, and recurrence rates of non-invasive auricular correction were compared among the four groups.

### Correction method

A non-invasive auricular corrector was used, which mainly includes an auricular base (with antihelix support), a helical retractor, a conchal cavity corrector, and an outer cover. The treatment steps are briefly described as follows:

1 Before use, prepare the skin by shaving off about two finger widths of hair around the ear. Clean the skin by using an alcohol swab to remove grease. Apply 3 M liquid dressing to the skin to form a colorless, transparent, and breathable protective film on the surface, effectively isolating the adhesive from direct skin contact.
2 Gently pull the upper 1/3 of the auricle that is buried under the temporal scalp, and shape the auricle, antihelix, antihelix crus, and scaphoid fossa according to the deformities. Try to stretch the skin as much as possible, and fix the auricular cartilage at the upper edge onto the correction model. When the auricular cartilage is hard, the muscle attachment behind the ear is short, or there is significant cartilage deficiency, adjust the position of the retractor according to individual circumstances. If it is difficult to correct all deformities at once, we opt for staged treatment. The entire process requires active parental involvement. After continuous use for one week, a follow-up visit is required. The parents should remove the orthotic device and dressings at home one day before the follow-up. If any discomfort occurs during the wearing period, patients can consult via WeChat, and the doctor will provide guidance based on the situation.
3 The treatment duration is determined based on the degree of deformity and the child's age. Generally, the treatment lasts for 1–3 months. During the treatment, the child should avoid strenuous activities and protect the auricle from external forces to prevent damage to the correction device.
4 After the treatment, the correction device should be removed, and the auricle should be observed for any changes. If the correction is effective, it can be continued to consolidate the effect. If the correction is not effective, the treatment can be continued, or other methods can be considered.
5 The success of the treatment is evaluated based on the improvement of the auricle's shape, the disappearance of the superior auricular sulcus, and the recovery of the auricular cartilage. The recurrence rate is also monitored to evaluate the long-term effectiveness of the treatment.

### Follow-up

All children were followed up for at least six months after treatment. Follow-up appointments could be made at the outpatient clinic, or follow-ups could be conducted via telephone or WeChat, depending on the specific circumstances.

### Efficacy evaluation criteria

① Effective: The auricle's shape basically returns to normal appearance, or there is some improvement compared to before correction, but it has not fully achieved a normal appearance; ② Ineffective: There is no improvement compared to before correction; ③ Recurrence: The auricle's shape improves and then rebounds to the uncorrected state.^5^

### Statistical analysis

Data analysis was processed using SPSS 27.0 statistical software. Qualitative indicators were described using rates; quantitative indicators were described using the mean ($\bar{x}$) ± Standard Deviation (s). Comparative analysis between two groups was conducted using Chi-Square tests or Fisher's exact probability method for qualitative data, and independent sample *t*-tests for quantitative data that followed a normal distribution between groups. For data that did not follow a normal distribution, the non-parametric rank sum test was used. All statistical tests were two-tailed, with *p* < 0.05 indicating a statistically significant difference, and *p* < 0.01 indicating a highly statistically significant difference.

## Results

In the study, children were divided into four groups based on age: A (less than 28 days), B (28–90 days), C (90–180 days), and D (greater than 180 days). The average age, number of children, and number of affected ears in each group are detailed in Table 1.

**Table 1:** General information of each group.

Group	Average age (days)	Number of children (cases)	Number of affected ears (ears)
A	25.29	7	8
B	54.15	20	24
C	105.44	9	9
D	224.67	6	7

### Gender distribution among groups

In terms of the children's gender, there were no statistically significant differences (*p* > 0.05) between any two groups (A, B, C, D) when analyzed using Fisher's exact probability method (Table 2).

**Table 2:** Gender distribution of children in each group (cases).

Group	Number of children	Male	Female
A	7	5	2
B	20	12	8
C	9	6	3
D	6	4	2

### Data on the location of affected ears

In terms of the location of the affected ear, there were no statistically significant differences between groups A, B, C, and D when pairwise comparisons were made using Fisher's exact probability test (*p* > 0.05) (Refer to Table 3).

**Table 3:** Distribution of affected ears by group (number of ears).

Group	Number of affected ears	Left ear	Right ear
A	8	3	5
B	24	10	14
C	9	4	5
D	7	2	5

### Comparison of treatment duration

After statistical analysis, the distribution of treatment duration in group B did not conform to a normal distribution. Pairwise comparisons were made using independent sample *t*-tests for those with normal distribution and rank sum tests for those without normal distribution. In terms of treatment duration, there were significant differences among groups A, C, and D (*p* < 0.01). There was no statistical difference between groups B and C (*p* > 0.05), but there were statistical differences between groups B and D, and between groups C and D (*p* < 0.05) (Refer to Table 4).

**Table 4:** Comparison of treatment duration among groups (mean ± SD) (days).

Group	Number of affected ears	Treatment duration
A	8	23.88 ± 1.94
B	24	32.96 ± 1.56
C	9	34.56 ± 1.73
D	7	42.57 ± 3.50

### Comparison of the efficiency of cartilage deformity correction

In terms of the efficiency of cartilage deformity correction, pairwise comparisons among groups A, B, C, and D using Fisher's exact probability method showed no statistical difference between groups A and B, but there were statistical differences between groups A and C, and A and D (*p* < 0.05) (Refer to Table 5).

**Table 5:** Effectiveness of cartilage deformity correction in each group (ears).

Group	Number of affected ears	Effective	Ineffective	Efficiency rate (%)
A	8	8	0	100
B	24	19	5	83.3
C	9	4	5	44.4
D	7	0	7	0

### Comparison of complications

In terms of complications, which mainly included rashes and skin damage, there were significant differences between groups A and C, A and D, and B and D (*p* <  0.01) after pairwise comparisons using Fisher's exact probability method. There was a statistical difference between groups B and C (*p* = 0.047; *p* < 0.05), but no statistical difference between groups A and B, and C and D (*p* > 0.05) (Refer to Table 6).

**Table 6:** Comparison of complications among groups (ears).

Group	Number of ears	With	Without	Incidence rate (%)
A	8	0	8	0
B	24	8	16	33.3
C	9	7	2	77.8
D	7	7	0	100

**Fig. 2:**
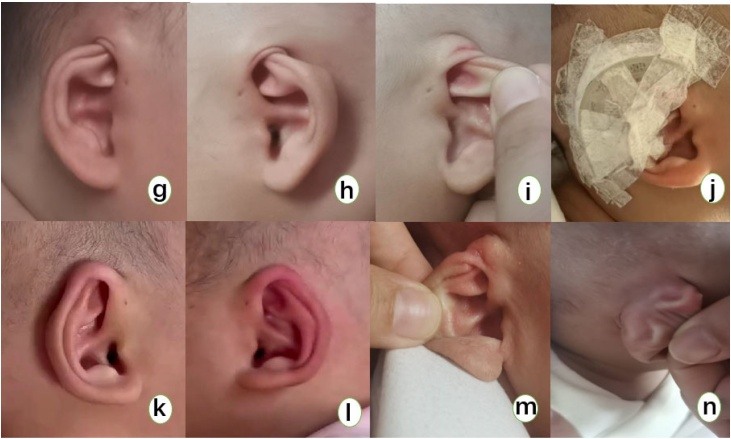
(Figures g‒n) Illustrate the correction process of a 2-month-old child with bilateral hidden ear deformity and cartilage malformation. (Figures g‒h) Show the upper part of the auricle cartilage buried under the temporal skin before correction. (Figure i) Shows the twisted and shortened cartilage that is buried. (Figure j) Illustrates the correction process of the hidden ear with cartilage malformation. (Figures k‒i) Show the corrected ear after one month of treatment with an auricular molding device. (Figure m) Shows the upper 1/3 of the auricle cartilage folding and collapsing backward. (Figure n) Shows the twisted cartilage behind the ear.

During the treatment process, all children achieved 100% effectiveness with non-invasive correction using ear splints. The upper part of the children's auricles that were embedded in the temporal subcutaneous tissue showed improvement compared to before the correction. As the children grew older and the treatment duration increased, the number of complications also increased. Groups A and B had high rates of effective correction of deformities, and the auricle shape was basically restored to normal appearance after cartilage correction (Fig. 2). There was no significant improvement in cartilage deformities in group D. After six months of observation, no recurrence of subcutaneous ear cartilage or cartilage deformity was observed in any of the four groups.

## Discussion

Infants with cryptotia are considered the fourth most common auricular deformity,^11^ and it is the type of auricular anomaly most easily detected by parents. The incidence rate of cryptotia among Japanese newborns is reported to be 0.2%.^11^ In China, there is currently no exact statistic on the incidence rate. Males are more frequently affected than females, with the right ear being more commonly involved than the left, and unilateral cases are more prevalent than bilateral cases,^12^, ^13^ which is consistent with the statistics from our study showing that boys account for 64.3%, the right ear for 60.4%, and unilateral cases for 85.7%.

The pathogenesis of cryptotia is not yet fully understood, and several possible causes have been proposed, including: (1) The genetic defect theory, which suggests that cryptotia may be an X-linked recessive trait^14^; (2) The cartilage developmental defect theory, which posits that cryptotia results from abnormal development of the auricular cartilage during embryonic life^15^; (3) The theory of anomalies in the development of the intrinsic muscles of the auricle.^15^ The most widely accepted theory at present is the auricular muscle abnormality theory. Yotsuyanagi et al.^16^ conducted anatomical studies on patients with congenital cryptotia and found that the most common cause of cryptotia is the shortening and abnormal insertion of the superior auricular muscle into the auricular cartilage, followed by the shortening of the oblique or transverse muscles of the auricle, with most patients having abnormalities in two or three muscles. Auricular muscles enable the movement and shape change of the auricle, but their function in humans has regressed, serving merely to maintain shape. Domestic researcher Wang Enyuan believes that cryptotia is mainly due to the superior auricular muscle's attachment point not being on the triangular fossa prominence at the back of the ear but instead attaching to the helix, causing the upper part of the auricle to become shallow and buried under the skin. Some cases are accompanied by the inward folding of the helix and the over-protrusion of the antihelix, primarily due to the wide attachment of the transverse muscle on both sides behind the antihelix.^17^

Previous reports on the treatment of cryptotia have largely focused on plastic surgery. For patients with mild cryptotia, after severing the adhesions on the auricular cartilage and the surrounding abnormally routed muscles, the originally mildly deformed auricular cartilage can be corrected, with only the issue of insufficient skin coverage after the auricle is lifted needing correction. For patients with severe auricular cartilage deformities, merely severing the abnormal adhesions and correcting the insufficient skin coverage after auricle lifting is still insufficient to completely solve the problem; other methods are needed to correct the auricular cartilage deformities. Surgical treatments have ranged from “V‒Y” flap advancement, local flap rotation, auricular cartilage expansion, to more recent approaches using conchal cartilage or other materials as support to correct the folding deformities of the antihelix and the subsequent upper one-third collapse and inversion of the auricle.^18^, ^19^ These methods primarily aim to solve the problem of creating the antihelix sulcus and providing additional skin coverage for the previously buried cartilage; additionally, they aim to correct cartilage defects and release abnormal intrinsic ear muscles.^4^ However, surgery carries risks such as insufficient postoperative expansion, retraction of the upper auricle, and complications like hematoma, infection, and scarring.^5^, ^19^ It is because of these issues associated with surgical treatment that non-invasive correction techniques are increasingly anticipated.

Patients with mild cryptotia typically exhibit auricular cartilage deformities characterized by: 1) A convergence deformity of the helix and scaphoid fossa; 2) A convergence of the superior and inferior crura of the antihelix, with the triangular fossa structure becoming unclear; 3) An angular deformity at the upper part of the helix, resulting in a pointed tip. In contrast, patients with severe cryptotia often present with additional deformities, including a twisted auricle, mainly manifested as the upper one-third of the auricle folding backward and collapsing^17^ (Fig. 2). In recent years, with the introduction of non-invasive auricular correctors into the Chinese market, numerous literature reports have demonstrated significant effects of non-invasive correction for mild cryptotia, with noticeable results even when treated at six months of age. However, there is currently a lack of research on severe cryptotia, and most clinicians still believe that children with congenital cryptotia accompanied by cartilage deformities are not suitable for non-invasive correction and should await later surgical treatment. Some scholars^10^, ^20^ have reported significant treatment effects of non-invasive correction in older children with cryptotia, with the buried auricular cartilage being successfully pulled out. However, none of them provided further classification and description of the cartilage deformities. Early studies by foreign scholars such as Hiroyasu Hirose^6^ also suggested that cryptotia in older children could still be corrected using non-invasive correction devices. Yet, none mentioned the effectiveness of non-invasive correction for cryptotia accompanied by cartilage deformities. Through our previous clinical research, we found that early intervention can treat cartilage deformities that trouble plastic surgeons through non-invasive correction, with significant effects. In this study, we found that the correction rate of cartilage deformities in groups A and B was significantly higher than in groups C and D. This indicates that non-invasive correction of cryptotia with cartilage deformities should be conducted within three months, with earlier correction leading to shorter treatment times and fewer treatment complications. Our study results show that after treatment with the non-invasive auricular molding device, all four groups of children achieved 100% effectiveness. The upper part of the auricle, which was buried under the temporal skin, improved compared to before correction in all children. This indicates that even in children older than 3-months, early non-invasive correction is effective as long as there is insufficient skin at the upper pole of the auricle.

**Fig. 3:**
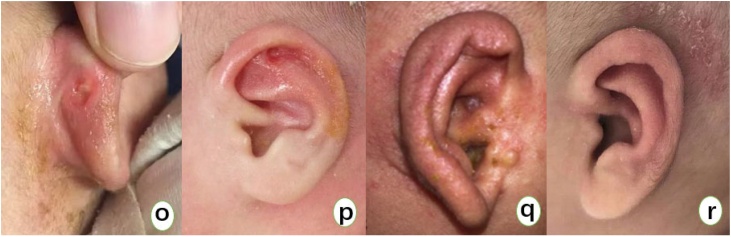
(Figure o) Shows skin damage behind the ear, mainly caused by the pad on the antihelix. (Figure p) Shows skin damage in the scapha, primarily due to the traction force of the device. Both conditions can improve with the application of antibiotic ointment locally. (Figure q) Shows a rash on the auricle, and (Figure r) shows a rash behind the ear. Both can improve with the application of eczema cream locally.

Third-degree cryptotia is characterized by a deficiency of skin at the upper pole of the auricle and underdeveloped cartilage. Non-invasive auricular correctors primarily address the insufficiency of skin and the deformities of the cartilage. During the correction process, when the skin of the upper auricle is tightly stretched and the cartilage deformity is severely twisted, we do not simply pull on the severely deformed areas, as this may lead to the traction device slipping, the development of local pressure sores, and a lack of noticeable correction effects. Instead, we fully utilize the normal cartilage above and below the deformed cartilage as a guide, while also using the push-pull effect of the ear support behind the ear, applying force front and back, and taking advantage of the plasticity of the child's local cartilage and skin for reshaping. From our research, it is understood that cartilage deformities show significant correction effects within three months, and partial correction is effective within six months. It can be said that if non-invasive auricular correctors are used for early treatment, they can greatly avoid the need for later surgical procedures in children, and are worth promoting among clinicians. Although the incidence of complications increases with age during correction, they are primarily rashes and skin damage (Fig. 3). As long as these issues are detected and treated promptly with medication, recovery is usually swift.

In summary, the non-invasive auricular correction for congenital cryptotia with cartilage deformities in infants and young children is significantly effective, with shorter correction times, higher correction rates for cartilage deformities, and fewer complications for those under three months old; older children can still consider non-invasive correction, but the correction time is relatively longer, and for those over six months old, the correction is still effective, but the rate of correction for cartilage deformities is lower, and there are more complications.

## Funding

This research did not receive any specific grant from funding agencies in the public, commercial, or not-for-profit sectors.

## Declaration of competing interest

The authors declare no conflicts of interest.
